# Folate, Vitamin B12 and Homocysteine status in the post-folic acid fortification era in different subgroups of the Brazilian population attended to at a public health care center

**DOI:** 10.1186/s12937-015-0006-3

**Published:** 2015-02-19

**Authors:** Aline Barnabé, Ana Cláudia Morandi Aléssio, Luis Fernando Bittar, Bruna de Moraes Mazetto, Angélica M Bicudo, Erich V de Paula, Nelci Fenalti Höehr, Joyce M Annichino-Bizzacchi

**Affiliations:** 1 Department of Clinical Pathology, Faculty of Medical Sciences, University of Campinas, Rua Alexander Fleming, 105, Campinas, SP 13083-881 Brazil; 2 Hematology and Hemotherapy Center, University of Campinas, Rua Carlos Chagas, 480, Campinas, SP 13083-878 Brazil; 3 Department of Pediatrics, Faculty of Medical Sciences, University of Campinas, Rua Tessália Vieira de Camargo, 126, Campinas, SP 13083-887 Brazil

**Keywords:** Folate, Vitamin B12, Homocysteine, Folic acid fortification, Reduced folate carrier, Glutamate carboxypeptidase II, Methylenetetrahydrofolate reductase

## Abstract

**Background:**

Folate and vitamin B12 are essential nutrients, whose deficiencies are considerable public health problems worldwide, affecting all age groups. Low levels of these vitamins have been associated with high concentrations of homocysteine (Hcy) and can lead to health complications. Several genetic polymorphisms affect the metabolism of these vitamins. The aims of this study were to assess folate, vitamin B12 and homocysteine status in distinct Brazilian individuals after the initiation of folic acid fortification by Brazilian authorities and to investigate the effects of RFC1 A80G, GCPII C1561T and MTHFR C677T polymorphisms on folate, vitamin B12 and Hcy levels in these populations.

**Methods:**

A total of 719 individuals including the elderly, children, as well as pregnant and lactating women were recruited from our health care center. Folate, vitamin B12 and Hcy levels were measured by conventional methods. Genotype analyses of RFC1 A80G, GCPII C1561T and MTHFR C677T polymorphisms were performed by PCR-RFLP.

**Results:**

The overall prevalence of folate and vitamin B12 deficiencies were 0.3% and 4.9%, respectively. Folate deficiency was observed only in the elderly (0.4%) and pregnant women (0.3%), whereas vitamin B12 deficiency was observed mainly in pregnant women (7.9%) and the elderly (4.2%). Plasma Hcy concentrations were significantly higher in the elderly (33.6%). Pregnant women carrying the MTHFR 677TT genotype showed lower serum folate levels (p = 0.042) and higher Hcy levels (p = 0.003). RFC1 A80G and GCPII C1561T polymorphisms did not affect folate and Hcy levels in the study group. After a multivariate analysis, Hcy levels were predicted by variables such as folate, vitamin B12, gender, age and RFC1 A80G polymorphism, according to the groups studied.

**Conclusion:**

Our results suggest that folate deficiency is practically nonexistent in the post-folic acid fortification era in the subgroups evaluated. However, screening for vitamin B12 deficiency may be particularly relevant in our population, especially in the elderly.

## Background

Folate and vitamin B12 are essential nutrients, and their deficiencies represent public health problems worldwide, affecting all age groups and leading to complications such as anemia, birth defects and neurological disorders [1,2]. Low concentrations of folate and vitamin B12 are also associated with high homocysteine (Hcy) levels, considered a risk factor for cardiovascular disease, cognitive decline and adverse pregnancy outcomes [2-4].

Folate deficiency can be caused by inadequate dietary intake, medications (methotrexate and anticonvulsants), alcoholism and conditions associated with increased cell turnover. In addition, a few genetic polymorphisms have been shown to influence folate levels. On the other hand, vitamin B12 deficiency results mainly from gastrointestinal conditions leading to vitamin B12 malabsorption, and less frequently from intestinal parasitosis and genetic polymorphisms [5].

Genetic polymorphisms such as reduced folate carrier (RFC1) A80G and glutamate carboxypeptidase II (GCPII) C1561T have also been shown to impair folate transport and absorption, respectively, thus affecting the bioavailability of the dietary folate [6,7]. The methylenetetrahydrofolate reductase (MTHFR) C677T polymorphism is associated with elevated Hcy levels and reduced folate and vitamin B12 levels [8,9].

Fortification of white flour with folic acid is mandatory in several countries and has proved to be a successful public health intervention. Although the major purpose of folic acid fortification is to reduce the occurrence of neural tube defects (NTD) during pregnancy, an additional benefit is a potential protection against chronic diseases, through its association with lower Hcy levels [10,11]. In Brazil, this strategy has been applied since 2004 by the National Health Surveillance Agency, ANVISA [12]. Fortification was implemented to provide an adequate intake of folate mainly for vulnerable populations, such as pregnant women, lactating women, children and the elderly [13,14].

In this context, the aims of this study were: (1) to assess folate and vitamin B12 status, as well as the frequency of elevated Hcy levels among the elderly, children, pregnant women and lactating women consecutively assisted at a public health care center after folic acid fortification; and (2) to investigate the effects of RFC1 A80G, GCPII C1561T and MTHFR C677 polymorphisms on folate, vitamin B12 and Hcy levels in the same study groups.

## Methods

### Study population

Seven hundred and nineteen participants were enrolled from April 2006 to May 2007, and divided into the following subgroups: 262 elderly individuals (≥60 year old), 106 children (≤6 year old), 291 pregnant women and 60 lactating women. The study group was chosen based on their vulnerability to vitamin deficiencies. We excluded individuals who had hypothyroidism, renal and hepatic disease, and those using agents that affect vitamin B metabolism such as methotrexate and anticonvulsants. In addition, individuals with acute illnesses were also excluded. Information regarding age, gender, height, weight, medical history, smoking, use of multivitamin supplements and gestational stage were collected.

The study groups were composed of individuals consecutively attended to at a public health care center in the northern area of the city of Campinas, Brazil, during visits to primary care physicians. Our recruitment goal was 250 individuals from each group, but due to the exclusion criteria and rate of refusal to participate this number could not be reached. Thus, all eligible participants, who were in accordance with the study protocol and who agreed to participate in the study, provided a written informed consent. For children, the written informed consent was obtained from mothers or guardians. The study protocol was approved by the Ethics Committee of the University of Campinas.

### Blood collection

Fasting blood samples were obtained from all participants in tubes without anticoagulant, for measurement of folate and vitamin B12, and in tubes containing EDTA to measure Hcy levels and for the genetic analyses (RFC1 A80G, GCPII C1561T and MTHFR C677T). Serum and plasma samples were processed within 3 hours of collection and stored below −80°C until analyses.

### Laboratory assays

Serum levels of folate and vitamin B12 were determined using chemiluminescence immunoassays (Elecsys/Roche Diagnostics, Mannheim, Germany). Hcy in plasma was assessed by high-performance liquid chromatography (HPLC) with fluorescence detection [15]. Genomic DNA was isolated from whole blood by standard methods. The analyses of RFC1 A80G, GCPII C1561T and MTHFR C677T polymorphisms were accomplished by PCR-RFLP. The amplified fragments were digested by restriction enzymes (*HinfI*, *HhaI* and *AccI*, respectively), as previously described [6,16,17].

We separated folate, vitamin B12 and Hcy levels into categories, based on the reference values used in our service. For folate levels, categories were: <4 ng/mL, ≥4 ng/mL; for vitamin B12: <200 pg/mL, 200 – 300 pg/mL, >300 pg/mL; and for Hcy levels, these categories were: <15 μmol/L, ≥15 μmol/L. Individuals with levels of folate below 4 ng/mL and vitamin B12 below 200 pg/mL were categorized as having deficiency. Vitamin B12 levels between 200 – 300 pg/mL were considered as marginal status [5,14]. Levels of Hcy ≥15 μmol/L were considered elevated [18]. It is noteworthy that the reference values used to describe folate and vitamin B12 deficiencies are similar to those cited by WHO [19].

### Statistical analysis

Data are expressed as median, percentiles 25 and 75, absolute number and percentages. Medians were compared using the Mann–Whitney test for 2-group comparisons and the Kruskal-Wallis test for 3-group comparisons. The Hardy-Weinberg equilibrium test was performed for all genotypes using the chi-square test. Multiple linear regression analysis with stepwise criteria was performed for Hcy as the dependent variable and folate, vitamin B12, polymorphisms, age, gender, body mass index, hypertension, diabetes and smoking as the independent variables. This analysis was adjusted for gestational stage in pregnant women. Statistical tests were performed using SPSS software for Windows version 17.0 (SPSS Inc, Chicago, IL, USA) and SAS System for Windows version 9.2 (SAS Institute Inc, Cary, NC, USA) with the level of significance set at p < 0.05.

## Results

During the enrollment period, all individuals who assisted at a public health care center were invited to participate in the study. A total of 719 individuals were included in our study. Among them, 40.5% were pregnant women, 36.5% were elderly, 14.7% were children and 8.3% were lactating women. The demographic characteristics of the studied population are summarized in Table 1.

**Table 1:** **Demographic characteristics of the study population**

	Elderly	Children	Pregnant women	Lactating women
	(N = 262)	(N = 106)	(N = 291)	(N = 60)
**Age (y)**	67 (60 – 91)	3 (0.5 - 6)	26 (14–43)	26.5 (14–40)
**Gender** (male)	114	54	-	-
**BMI** (mean ± SD)	26.7 (23.9, 29.9)	15.9 (14.8, 17.9)	26.4 (23.4, 30.7)	23.4 (21.5, 26.4)
**Supplementation**^**1**^ [n (%)]	43 (16.4)	22 (16.6)	138 (47.4)	10 (16.6)
**Smoking** [n (%)]	20 (7.6)	-	27 (9.3)	8 (13.3)
**Hypertension**^**2**^ [n (%)]	158 (60.3)	-	42 (14.4)	5 (8.3)
**Diabetes** [n (%)]	65 (24.8)	3 (2.8)	12 (4.1)	1 (1.6)
**Dyslipidemia** [n (%)]	65 (24.8)	-	2 (0.7)	-
**Gestational stage**^**3**^ n (%)]				
First trimester	-	-	73 (27.8)	-
Second trimester	-	-	125 (47.5)	-
Third trimester	-	-	65 (24.7)	-

Age is expressed as median and range in parentheses. Body Mass Index (BMI) is expressed as median and percentiles (25 and 75) in parentheses. Gender is expressed as number of individuals.

^1^Users of multivitamins or folic acid supplements.

^2^Hypertension definition: blood pressure exceeding 140 over 90 mmHg.

^3^Gestational stage was not available for 28 pregnant women.

### Folate, vitamin B12 and homocysteine levels

Among the distinct study groups, children had higher vitamin B12 levels, the elderly had higher Hcy levels and lactating women had lower folate levels (Table 2).

**Table 2:** **Folate, vitamin B12, and Hcy levels in different study groups**

Groups	Folate(ng/mL)	Vitamin B12(pg/mL)	Hcy ^ 1 ^ (μmol/L)
**Elderly**	11.2(8.7, 13.6)	443(333, 620.2)	13.5(11.1, 17.1)
	N = 262	N = 262	N = 262
**Children**	12.4(9.4, 14.6)	853.0(611, 1188)	6.2(5.2, 7.3)
	N = 103	N = 103	N = 105
**Pregnant women**	10.7(8.3, 14.1)	325.0(257, 424)	6.4(5.3, 7.5)
	N = 291	N = 291	N = 291
**Lactating women**	9.8(7.6, 12.2)	523.0(415.7, 641.5)	9.2(7.6, 10.8)
	N = 54	N = 54	N = 60
***P*** ^**2**^	0.003	p < 0.001	p < 0.001

All values are expressed as median and percentiles (25 and 75).

^1^Hcy, homocysteine.

^2^Kruskal-Wallis test.

The overall frequency of folate and vitamin B12 deficiencies were estimated to be 0.3% and 4.9% respectively (Table 3). Interestingly, we observed no folate or vitamin B12 deficiencies in the children included in our study, and only 1% presented marginal status of vitamin B12. Folate deficiency could be identified in 0.4% of elderly and 0.3% of pregnant women. None of the lactating women showed folate deficiency. The pregnant women group was the one which presented a higher frequency of vitamin B12 deficiency (7.9%), followed by the elderly (4.2%) and lactating women (1.9%). Marginal status of vitamin B12 was observed in 14.5% of the elderly, 33.7% of pregnant women and 7.4% of lactating women. The frequency of elevated Hcy levels was observed mainly in the elderly (33.6%). Among pregnant and lactating women, the frequencies of elevated Hcy levels were 0.7 and 5.0%, respectively.

**Table 3:** **Folate, vitamin B12 and Hcy status according to cut-off values**

	Cut-off values	All	Elderly	Children	Pregnant women	Lactating women
		N, %	N, %	N, %	N, %	N, %
**Folate**						
	<4 ng/mL	2, 0.3	1, 0.4	-	1, 0.3	-
	≥4 ng/mL	708, 99.7	261, 99.6	103, 100	290, 99.7	54, 100
**Vitamin B12**						
	<200 pg/mL	35, 4.9	11, 4.2	-	23, 7.9	1, 1.9
	200 - 300 pg/mL	141, 19.9	38, 14.5	1, 1.0	98, 33.7	4, 7.4
	>300 pg/mL	534, 75.2	213, 81.3	102, 99.0	170, 58.4	49, 90.7
**Homocysteine**						
	<15 μmol/L	625, 87.0	174, 66.4	105, 100	289, 99.3	57, 95.0
	≥15 μmol/L	93, 13.0	88, 33.6	-	2, 0.7	3, 5.0

### Effects of polymorphisms RFC1 A80G, GCPII C1561T and MTHFR C677T in the studied population

In order to assess the effects of polymorphisms on folate, vitamin B12 and Hcy levels we compared the median of these parameters in individuals with different genotypes (Table 4). Folate, vitamin B12 and Hcy concentrations were not affected by the RFC1 A80G, GCPII C1561T and MTHFR C677T polymorphisms in the elderly, children and lactating women. Pregnant women carrying the MTHFR 677TT genotype showed lower serum folate levels (p = 0.042) and higher Hcy levels (p = 0.003).

**Table 4:** **Folate, vitamin B12 and Hcy levels according to different polymorphisms in the study groups**

	GROUPS	RFC1 A80G ^ 3 ^			*P* ^ 1 ^	GCPII C1651T ^ 4 ^		*P* ^ 2 ^	MTHFR C677T ^ 5 ^			*P* ^ 1 ^
		AA	AG	GG		CC	CT		CC	CT	TT	
**Folate (ng/mL)**												
	**Elderly**	11.2 (8.4,13.2)	11.2 (8.8, 13.9)	11 (8.6, 13.2)	0.840	11 (8.5, 13.7)	12.2 (11.1, 13)	0.252	11.2 (8.6, 13.3)	11 (8.8, 14.2)	10.2 (7.3, 12.4)	0.158
		N = 49	N = 125	N = 86		N = 241	N = 14		N = 103	N = 118	N = 36	
	**Children**	12.5 (8.2,16.1)	12.5 (9.4, 15.1)	12.5 (10.2, 14.1)	0.863	12 (9.4, 15.1)	11.5 (8.2, 18.3)	0.932	12.0 (9.3, 14.4)	12.5 (9.2, 14.9)	13.8 (9.6, 15.6)	0.441
		N = 20	N = 60	N = 20		N = 79	N = 6		N = 51	N = 32	N = 11	
	**Pregnant women**	9.8 (8.0,13.6)	11 (8.4, 15)	10.9 (8.5, 14.2)	0.346	10.8 (8.2, 14.3)	11.5 (8.5, 13.3)	0.927	11.2 (9, 15)	10.8 (7.7, 14)	8.8 (7.3, 11.6)	0.042
		N = 74	N = 129	N = 83		N = 250	N = 21		N = 139	N = 113	N = 26	
	**Lactating women**	10.6 (9.4,12.7)	9.5 (7, 12.1)	9.5 (7.3, 11.7)	0.300	9.8 (7.7, 12)	12.6 (7.5, 14.3)	0.429	10 (9.1, 12.5)	9.3 (7.1, 13.1)	9.7 (6.8, 10.8)	0.723
		N = 12	N = 28	N = 14		N = 49	N = 3		N = 27	N = 21	N = 4	
**Vitamin B12 (pg/mL)**												
	**Elderly**	418 (310, 687.5)	446 (341, 623)	442 (337.5, 584.7)	0.650	444 (333, 616.5)	373.5 (288.2, 647.7)	0.317	463 (345, 594)	431 (330.5, 627.5)	391.5 (276.2, 577.5)	0.443
		N = 49	N = 125	N = 86		N = 241	N = 14		N = 103	N = 118	N = 36	
	**Children**	827 (638.5, 1047.5)	874 (650.2, 1200)	770.5 (513, 1207.2)	0.840	901 (611, 1204)	772.5 (518.7, 1000.5)	0.359	790 (572, 1109)	918.5 (660.2, 1206)	756 (553, 1049)	0.333
		N = 20	N = 60	N = 20		N = 79	N = 6		N = 51	N = 32	N = 11	
	**Pregnant women**	341.5 (267.7, 462)	324 (253, 419)	319 (240, 412)	0.218	322 (253.7, 424)	324 (263.5, 399.5)	0.738	321 (249, 420)	334 (257.5, 425)	308.5 (236.2, 441.7)	0.703
		N = 74	N = 129	N = 83		N = 250	N = 21		N = 139	N = 113	N = 26	
	**Lactating women**	563 (470, 648)	467 (400.2, 540.7)	624 (408.7, 682)	0.114	488 (414, 622.5)	627 (576, 699)	0.094	488 (437, 627)	485 (377, 551.5)	617 (520, 712)	0.137
		N = 12	N = 28	N = 14		N = 49	N = 3		N = 27	N = 21	N = 4	
**Homocysteine (μmol/L)**												
	**Elderly**	14.1 (11.7, 17.9)	12.9 (10.7, 16.4)	13.8 (11.4, 17.2)	0.170	13.5 (11.2, 17.1)	13.3 (10.7, 18.4)	0.896	13.2 (10.9, 15.7)	13.3 (10.8, 17.2)	14.8 (12.1, 18.4)	0.087
		N = 49	N = 125	N = 86		N = 241	N = 14		N = 103	N = 118	N = 36	
	**Children**	6.0 (5.3, 6.7)	6.3 (5.1, 7.4)	6.7 (5.2, 7.4)	0.582	6.2 (5.2, 7.1)	5.9 (5.2, 8.4)	0.876	6.2 (5.4, 7.2)	5.8 (4.9, 7.5)	6.8 (6.5, 8)	0.099
		N = 20	N = 60	N = 20		N = 78	N = 6		N = 51	N = 32	N = 11	
	**Pregnant women**	5.9 (5.1, 7.6)	6.4 (5.4, 7.6)	6.3 (5.5, 7.5)	0.151	6.4 (5.4, 7.5)	6.2 (5.1, 7.8)	0.710	6.2 (5.4, 7.4)	6.3 (5.2, 7.3)	7.8 (6.3, 9.2)	0.003
		N = 74	N = 129	N = 83		N = 250	N = 21		N = 139	N = 113	N = 26	
	**Lactating women**	9.4 (8.2, 10.4)	9.7 (7.4, 11.5)	8.7 (7.8, 10.9)	0.929	9.4 (7.5, 10.8)	12.6 (8.9, 12.6)	0.193	9.4 (7.6, 10.7)	9.9 (8, 11.9)	7.8 (6.8, 11.4)	0.529
		N = 12	N = 28	N = 14		N = 49	N = 3		N = 27	N = 21	N = 4	

Values are expressed as median and percentiles (25 and 75).

^1^Kruskal-Wallis test.

^2^Mann-Whitney test.

^3^AA, wild type; AG, heterozygous; and GG homozygous mutant for the RFC1 A80G polymorphism.

^4^CC, wild type; and CT, heterozygous for the GCPII C1561T polymorphism.

^5^CC, wild type; CT, heterozygous; and TT, homozygous mutant for the MTHFR C677T polymorphism.

Homozygosity for the GCPII C1561T polymorphism was not found in our population.

Genotype distributions of RFC1 A80G, GCPII C1561T and MTHFR C677T polymorphisms were in Hardy-Weinberg equilibrium. Table 5 summarizes the frequencies of genotypes for each polymorphism studied.

**Table 5:** **Overall genotype frequencies of RFC1 A80G, GCPII C1561T and MTHFR C677T polymorphisms**

Polymorphisms	Genotypes	Study groups	
		N	%
**RFC1 A80G** ^**1**^			
	**AA**	158	22.3
	**AG**	343	48.4
	**GG**	207	29.3
**GPCII C1561T** ^**2**^			
	**CC**	619	93.4
	**CT**	44	6.6
**MTHFR C677T** ^**3**^			
	**CC**	326	47.3
	**CT**	286	41.5
	**TT**	77	11.2

^1^AA, wild type; GA, heterozygous; and GG homozygous mutant for the RFC1 A80G polymorphism.

^2^CC, wild type; and CT, heterozygous for the GCPII C1561T polymorphism.

^3^CC, wild type; CT, heterozygous; and TT, homozygous mutant for the MTHFR C677T polymorphism.

### Impact of clinical and laboratory parameters on Hcy levels

We next evaluated the impact of clinical and laboratorial parameters on Hcy levels (Table 6). Using a multiple linear regression analysis with stepwise criteria, the variables independently associated with Hcy levels were: folate, vitamin B12, gender, age and RFC1 A80G polymorphism (genotype AA) in the elderly; vitamin B12 in children; and folate in pregnant women. None of the variables evaluated showed any impact on Hcy levels in lactating women.

**Table 6:** **Predictors of Hcy levels in the elderly, children and pregnant women**

Groups	Independent variables	β	R ^ 2 ^	*P*
**Elderly**				
	Folate	−4.594	0.0441	p < 0.001
	Vitamin B12	−0.044	0.0324	p < 0.001
	Gender (male)	52.785	0.1622	p < 0.001
	Age	3.219	0.0716	p < 0.001
	RFC1 (AG x AA)	−26.995	0.0192	0.011
	(GG x AA)	−11.736		
**Children**				
	Vitamin B12	−0.023	0.0782	0.011
**Pregnant women**				
	Folate	−4.556	0.0750	p < 0.001

Multiple linear regression analysis with stepwise criteria. Independent variables: age, gender (male versus female), smoking, hypertension, diabetes, dyslipidemia, BMI, polymorphisms (RFC1 A80G, GCPII C1561T e MTHFR C677T), folate and vitamin B12. In pregnant women, the analysis was adjusted for gestational stage.

## Discussion

The present study suggests that fortification of flour with folic acid has been effective, as folate deficiency was practically nonexistent (0.3%), whereas vitamin B12 deficiency was present in 4.9% of the studied group. A previous study performed with Brazilian adults reported that folate deficiency was not detected and vitamin B12 deficiency was over 6% [20]. Similarly, folate deficiency is practically nonexistent and vitamin B12 deficiency is approximately 5% in the Canadian population [11]. In developed countries, folate deficiency is uncommon; however this deficiency can still be observed in some developing countries [21]. In addition, the prevalence of folate and vitamin B12 deficiencies vary between different studies due to differences in the assays employed [13].

We believe that our results represent the profile of these vitamins in the investigated group, as the measurements were performed in 719 individuals of various ages under several clinical conditions using laboratory methods considered suitable for analysis. Furthermore, the subgroup-specific evaluation is also important, enabling the identification of individuals at an increased risk of developing vitamin deficiencies. These individuals should be considered for specific prophylactic measures, as a problem in clinical practice is that sometimes the deficiencies are identified only when complications, such as anemia, NTD, and neurological disorders, have already occurred. Thus, prevention of folate and vitamin B12 deficiencies becomes a major challenge for health worldwide.

As described previously, folate deficiency may occur at any age, mostly in individuals ingesting a poor diet or suffering from intestinal malabsorption [22]. Moreover, vitamin B12 levels frequently decrease with age due to malabsorption of vitamins from food, which is more common in the elderly [11]. Approximately 10% of the elderly are estimated to present reduced levels of vitamin B12, with this prevalence increasing approximately 5% at the age of 65 years and to 20% at the age of 85 years [13,18]. In our study, we identified 0.4% and 4.2% of the elderly with folate and vitamin B12 deficiencies respectively, whereas 14.5% showed marginal levels of vitamin B12. Coussirat *et al.* evaluated 545 Brazilian elderly individuals, and detected 0.5% with folate deficiency, 5.5% with vitamin B12 deficiency, while 23.3% had marginal levels of vitamin B12. Xavier *et al.* showed that 7.2% of the elderly had a vitamin B12 deficiency [20,23].

Our results show a situation that can often go unnoticed in the elderly, which is a vitamin B12 deficiency. Atrophy of the gastric mucosa, the presence of auto-antibodies against intrinsic factors (often undiagnosed), or the presence of *H. pylori* may play a role as an etiological factor of vitamin B12 deficiency in this age group, because it results in malabsorption of vitamin B12.

Although almost all of these individuals in our study showed no anemia (data not shown), it should be emphasized that symptoms such as depression, dementia and impaired cognitive function, which have been associated with vitamin B12 deficiency, may be misinterpreted as aging-related co-morbidities rather than vitamin B12 dependent co-morbidities [24-26]. In this sense, our findings have great relevance in clinical practice, suggesting that the measurement of this vitamin should be part of routine diagnosis in patients over 60 years, even in the absence of hematological symptoms. The high frequency of elderly individuals with marginal levels of vitamin B12 is another important fact, although the clinical significance of these levels on their health are not clear [27].

Children aged from 0.5 – 6 years, included in our study, did not exhibit folate and vitamin B12 deficiencies. Several studies conducted in developing countries, such as Colombia, have described a very low prevalence of deficiency of these vitamins [28]. In Brazil, a study that included 1111 Amazonian children reported a folate and vitamin B12 deficiency of 2.5 and 3.7%, respectively [29]. Another study of 164 Brazilian children showed that deficiency of folate and vitamin B12 were present in 2.2 and 11.7%, respectively. It is important to note that this study included children under 2 years of age, who continued to receive cow’s milk and porridge or did not consume vegetables, fruits, and animal products until over the age of one; a fact that might have contributed to the high prevalence of vitamin B12 deficiency [30]. Shakur *et al.* showed that since food fortification with folic acid, folate deficiency has been reduced to practically 0% in children aged 1 – 14 years in Canada [31]. These results suggest that there is apparently no additional advantage of supplementation with folic acid in countries where food products are folic acid-fortified [14].

Our results suggest that fortification was adequate to prevent folate deficiency in these children, and that diet was capable of meeting vitamin B12 requirements. Vitamin B12 deficiency in children is exceptional as daily requirements are very small, and children normally consume food that contains this nutrient.

During pregnancy, folate and vitamin B12 are essential for normal fetal development. Furthermore, pregnant women have an increased physiological need for these nutrients, and their inadequate intake increases the risk of developmental abnormalities, including NTD [32,33]. The results of the present study showed that the median of folate (10.7 ng/mL) and vitamin B12 (325.0 pg/mL) in pregnant women were higher than those reported by Guerra-Shinohara *et al.* (5.6 ng/mL and 181.1 pg/mL, respectively) before folic acid fortification in Brazil [34].

We highlight that folate deficiency during pregnancy was virtually nonexistent in our study. According to some studies, the mandatory fortification of flour with folic acid resulted not only in an improvement in input and levels of folate in the blood, but also in a reduction in the occurrence of NTD [35-37]. Despite the lack of data on the outcome of these pregnancies, our results corroborate the effectiveness of this program, since normal folate levels were present in all pregnant women, irrespective of folate supplementations. However, we cannot exclude that supplementation may have contributed to achieving adequate folate levels in some of these pregnant women.

Vitamin B12 deficiency (7.9%) or marginal levels (33.7%) were frequent in the group of pregnant women. A high prevalence of this deficiency in this group has also been described in other populations [38]. Ray *et al.* suggested that 1 in 20 Canadian women may be vitamin B12 deficient during the critical period of closing the embryonic neural tube [39]. Increased risk of NTD has also been associated with vitamin B12 deficiency, especially after the fortification of flour with folic acid [40,41].

In the current study, practically all pregnant women with vitamin B12 deficiency were in their second and third trimester of pregnancy. It is known that vitamin B12 decreases through gestation due to an increase in fetal requirements [34,42,43], and therefore, the deficiency in this group should be interpreted with caution. In addition, we cannot rule out the effect of hemodilution upon the levels of vitamin B12 in pregnant women. Moreover, the cut-off used to identify the vitamin B12 deficiency in the general population may not apply during pregnancy.

In lactating women, a higher folate intake is also required. Folate concentration in human milk is strongly regulated and not affected by maternal folate status, except in clinically folate-deficient mothers [14]. Maternal vitamin B12 levels have also been correlated with milk vitamin B12_,_ and infant urinary methylmalonic acid levels, inversely related to milk vitamin B12 levels. However, in breast-fed infants the deficiency may become clear due to the low milk concentration of vitamin B12 [44,45]. In this study, we did not observe folate deficiency in lactating women; however 1.9% showed vitamin B12 deficiency.

Although the main purpose of fortifying flour with folic acid is the reduction of NTD, the potential benefit of reducing the risk of cardiovascular disease by reducing the levels of Hcy is also relevant [10]. Hyperhomocysteinemia has been considered a risk factor for vascular diseases and its increase is related to folate and vitamin B12 deficiencies, and genetic polymorphisms.

Some population-based studies suggest that a decline in mortality related to strokes coincided with the introduction of folic acid fortification in the United States and Canada [46]. However, some meta-analysis studies that evaluated the risk of cardiovascular disease or death in patients with or without previous disease, failed to show any beneficial effect of this strategy. Apparently, for some subgroups of patients, such as those with kidney disease, supplementation may have a beneficial effect [47,48].

Our results showed a higher frequency of hyperhomocysteinemia, mainly in the elderly. Hyperhomocysteinemia may be a consequence of ageing, oxidative stress, hypertension, diabetes and dyslipidemia. These factors were common in this group and could justify the high frequency of this condition. Moreover, our results corroborate with studies that consider the reduction of folate and vitamin B12 and age as factors related to hyperhomocysteinemia [49,50].

On the other hand, hyperhomocysteinemia was nonexistent in all the children included, and multivariate analysis demonstrated the effect of vitamin B12 levels. A study performed in 207 children from the region of Campinas demonstrated that acquired factors, vitamin B12 and folate, were the most important factor in defining the levels of plasma Hcy [51].

We have also observed that Hcy levels were normal in most of the pregnant women (99.3%). Hcy levels are known to be lower in pregnant women than in non-pregnant women [52,53]. However, studies have reported that elevated Hcy levels (>15.33 μmol/L) were observed in mothers of infants with NTD [54,55]. Furthermore, several studies have also associated high levels of Hcy to a variety of adverse effects during pregnancy [56].

In lactating women, hyperhomocysteinemia was present in 5%. Ramlau-Hansen *et al.* demonstrated that breastfeeding mothers who did not take folic acid supplements had a higher prevalence of elevated Hcy, compared to breastfeeding mothers taking folic acid supplements, and to a control population [57]. Despite this relationship between folate and hyperhomocysteinemia in lactating women, the multivariate analysis showed no factor that might interfere with Hcy levels, and this fact can be attributed to the number of participants included.

Among the polymorphisms investigated, only MTHFR C677T was significantly associated with folate and Hcy levels in pregnant women. Folate status is well-known to play a crucial role in the development of hyperhomocysteinemia in individuals with this polymorphism [58,59]. Furthermore, Hcy levels tend to be independent of the genotype in populations having high T allele frequency (≥20%) [60,61].

No interactions of the RFC1 A80G and GCPII C1561T polymorphisms were found on folate and Hcy levels, corroborating with previous studies. The effects of polymorphisms in folate-metabolizing genes on Hcy levels may be masked by the interaction with other polymorphisms or with environmental factors that influence folate status [62].

The frequencies of MTHFR C677T and RCF1 A80G genotypes were similar to those reported in others populations [17,62], whereas genotype frequencies of the GCPII C1561T polymorphism were different from those described in other studies [6,7].

Finally, we were able to demonstrate that Hcy levels are influenced mainly by folate and vitamin B12. Plasma Hcy may serve as an indicator of status and perhaps of the intake of vitamins such as folate and vitamin B12 [49]. These results also corroborate with previous findings where acquired factors contributed more to hyperhomocysteinemia than genetic factors [51].

In Brazil, a few studies have evaluated vitamin deficiencies after folic acid fortification, mainly in the population investigated and, therefore, we considered this analysis of an impressive number of more than 700 participants as the strength of the current study. However some limitations should be acknowledged, such as: the fact that our population is not representative of the entire country’s population which presents great diversity; the small number of children and lactating women included; plasma Hcy samples were not kept on ice until separation, and the length of time between the blood collection and plasma preparation could have a significant impact on plasma total Hcy; the lack of data from the pre-fortification era to compare with the post-fortification era; and also the lack of data regarding food-intake to correlate with folate and vitamin B12 levels. Also, we did not perform the measurements of red blood cell folate and methylmalonic acid. We could not exclude that the real impact of fortification was compromised in lactating women because this group comprised of only 60 individuals. These limitations should be considered when analyzing our data.

## Conclusion

Folate deficiency is practically nonexistent in the post-folic acid fortification era in the studied population. However, our study suggests that screening for vitamin B12 deficiency may be particularly relevant, especially in the elderly, and the impact of the relatively high frequency of this deficiency on the overall health of our population deserves additional studies. Regarding the influence of genetic polymorphisms, we observed no evidence that RFC1 A80G and GCPII C1561T polymorphisms-influenced folate, vitamin B12 and Hcy. Finally, we confirmed that folate and vitamin B12 are important determinants of Hcy levels.

## Abbreviations

ANVISA: National Health Surveillance Agency
GCPII: Glutamate carboxypeptidase II
Hcy: Homocysteine
HPLC: High-performance liquid chromatography
MTHFR: Methylenetetrahydrofolate reductase
NTD: Neural tube defect
RFC1: Reduced folate carrier
